# Near‐Complete tracheal obstruction due to mucormycosis: A report of two cases

**DOI:** 10.1002/ccr3.6278

**Published:** 2022-08-24

**Authors:** Mohammad Javad Fallahi, Reza Nikandish, Bizhan Ziaian, Reza Shahriarirad

**Affiliations:** ^1^ Thoracic and Vascular Surgery Research Center Shiraz University of Medical Sciences Shiraz Iran; ^2^ Department of Internal Medicine Namazi Hospital, Shiraz University of Medical Sciences Shiraz Iran; ^3^ Anesthesiology and Critical Care Research Center Shiraz University of Medical Sciences Shiraz Iran; ^4^ Department of Surgery Shiraz University of Medical Sciences Shiraz Iran; ^5^ Student Research Committee Shiraz University of Medical Sciences Shiraz Iran

**Keywords:** bronchoscopy, case report, diabetes mellitus, mucormycosis, tracheal

## Abstract

We present two cases with diabetes and mucormycosis of the major airways. Both patients underwent fiberoptic bronchoscopic evaluation, showing near‐complete occlusion of major airways with creamy necrotic mass lesions. Prompt and accurate diagnosis is vital to limit the extent of tissue destruction and prevent death due to asphyxia.

## Graphical abstract

Initial bronchoscopy demonstrating (A) obstructing creamy necrotic mass in the distal part of the trachea, carina, and proximal part of right main bronchus; (B) Gross view of the creamy necrotic mass diagnosed as mucormycosis; (C) follow up bronchoscopy 2 months later: main carina deformity and severe right main bronchus narrowing.

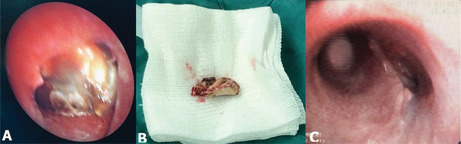

## 1 BACKGROUND

Mucormycosis is an invasive fungal infection that most commonly affects diabetic and immunocompromised patients, particularly in its rhino‐orbital‐cerebral or pulmonary forms. Rarely, mucormycosis may involve the trachea and main bronchi.^1^ Although pulmonary mucormycosis has been reported previously, few reports describe tracheal involvement by this fungus presenting with near‐fatal tracheal obstruction and remedied by an emergency bronchoscopic intervention.

## 2 CASE PRESENTATION

### 2.1 Case 1

A 37‐year‐old diabetic man was admitted to a medical ward with severe pneumonia and uncontrolled blood sugar. He presented with fever, dyspnea, stridor, and high blood sugar for 5 days before hospitalization. He was on prednisolone and azathioprine due to primary biliary cirrhosis 6 months before this hospital admission, along with metformin due to iatrogenic diabetes 2 months before his current admission. Chest examination revealed bilateral wheezing and rhonchi. Arterial blood gas studies were acceptable during the early hospital course. Computed tomography (CT) scan of the chest and mediastinum showed diffuse bilateral infiltration in the lower lobes of both lungs. Initially, broad‐spectrum antibiotics (intravenous imipenem and co‐trimaxazol for coverage of bacterial and possible pneumocystis jirovecii‐pneumonia) and insulin were administered; however, he had no favorable response and ultimately developed respiratory failure and underwent endotracheal intubation and mechanical ventilation and was transferred to the intensive care unit (ICU).

The patient was extubated 1 day after ICU ward admission. At this time, he was completely well apart from hoarseness in speech. Twenty‐four hours later, in the early morning, the patient developed severe respiratory distress associated with inspiratory stridor. Arterial blood gases showed a PaCo2 of 160 mmHg; therefore, re‐intubation was attempted. However, it was impossible to pass the endotracheal tube (ETT) deep enough through the larynx since there was significant resistance, so the ETT was fixed at 19 cm from the lips. Despite these measures, it was not possible to ventilate the patient even with high inspiratory pressures. The patient subsequently developed bradycardia, and therefore, emergency fiberoptic bronchoscopy was performed through the ETT after administration of intravenous atropine to prevent cardiac arrest.

**FIGURE 1:**
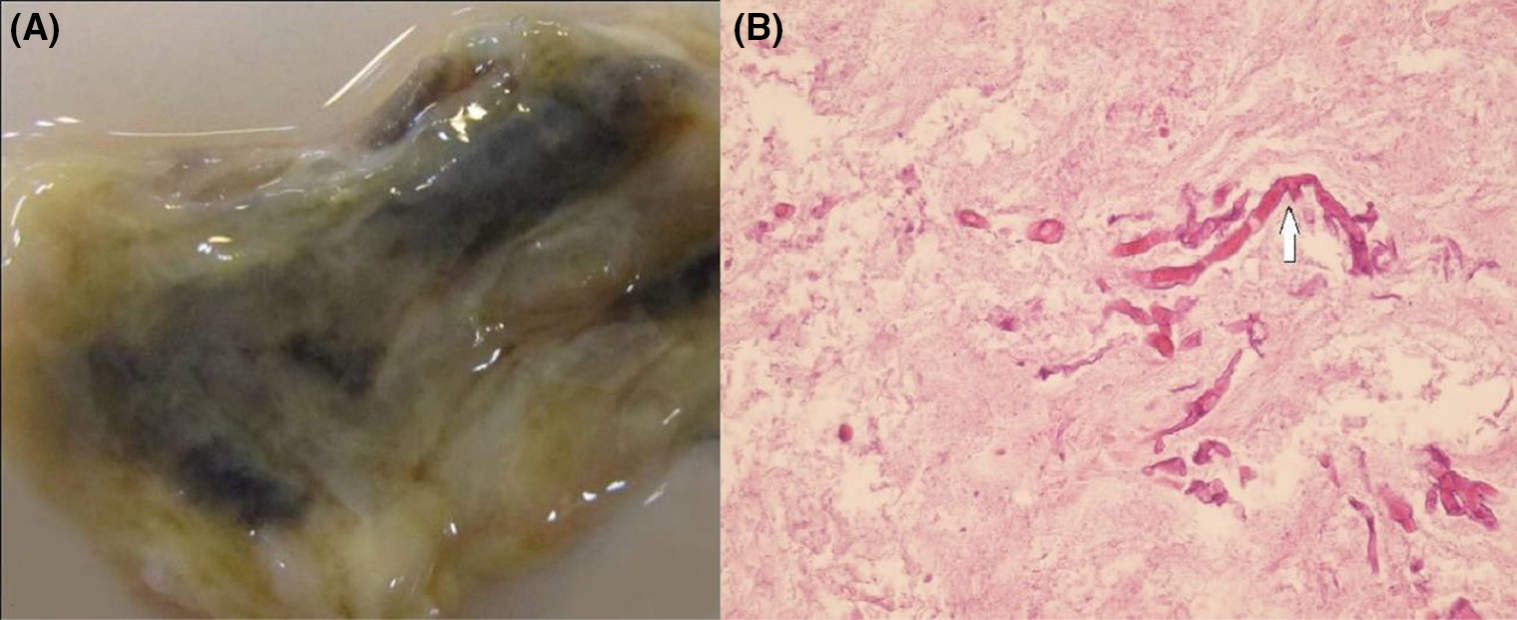
(A) soft and grayish‐white mass with a black core, which was removed from the trachea by rigid bronchoscopy; (B) Histopathology of the tracheal mass showing broad hyphae with right‐angle branching and no septation (arrow) consistent with mucormycosis

**FIGURE 2:**
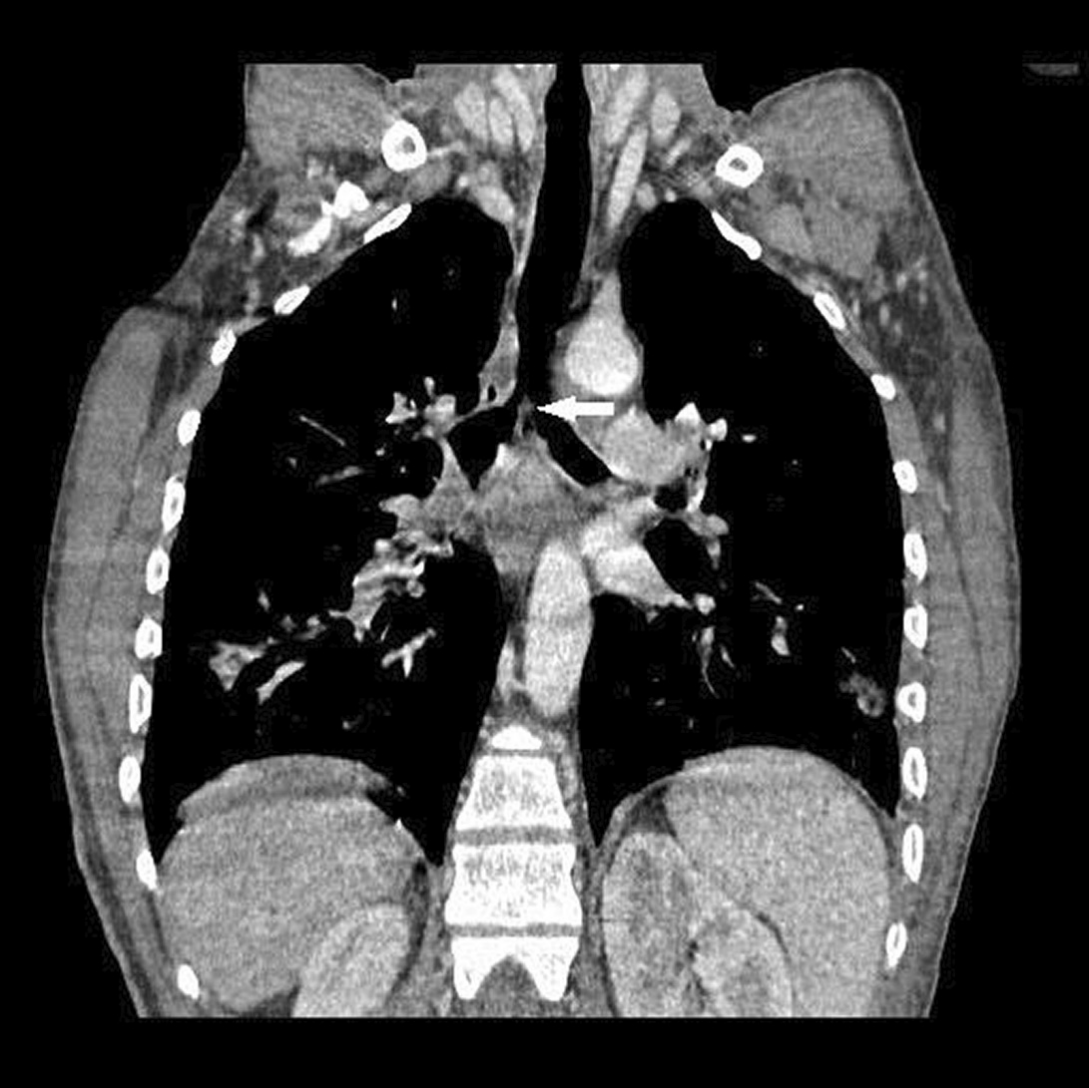
Coronal reconstructed CT image of the chest showing narrowing of distal trachea and main bronchi. A residual fungus mass on the carina (arrow) is seen

During fiberoptic bronchoscopy, the tracheal lumen was nearly completely obstructed with a creamy necrotic mass extending from the trachea's middle part to the carina's level. Through the bronchoscope's working channel, the mass was grasped with biopsy forceps; however, separation from the tracheal wall was impossible. Therefore, the mass was dissected into smaller fragments and removed from the trachea in several attempts. After removing a significant part of the intra‐luminal mass, ventilation of the patient was achieved with acceptable tidal volumes. After stabilizing the patient, the patient was scheduled for an emergency rigid bronchoscopy. Under general anesthesia, rigid bronchoscopy was done, and the obstructing mass was removed (Figure 1A). Contrast‐enhanced spiral chest CT scan was done the day after rigid bronchoscopy, which showed residual narrowing of the lower part of the trachea extending to the carina and main bronchi (Figure 2). After 2 days of intubation and mechanical ventilation, the patient was extubated in a good general condition. A histopathological study of the mass showed broad hyphae with right‐angle branches without septation, which were diagnostic for mucormycosis (Figure 1B).

Liposomal amphotericin B (5 mg/kg/day) was administered, and other antibiotics were discontinued. He was transferred to the medical ward to complete the course of antifungal treatment, which was completed after 30 days of hospital admission.

### 2.2 Case 2

The second patient was a 20‐year‐old man, known case of diabetes mellitus type one and glucose‐6‐phosphate dehydrogenase deficiency, who was hospitalized because of acute‐onset respiratory distress for one night before admission.

**FIGURE 3:**
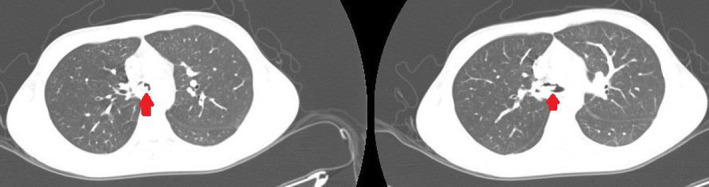
A computed tomography image of the second patient at the distal trachea and carina depicts severe tracheal narrowing and obstructing fungal mass in the right main bronchus (red arrow)

He had a history of hospitalization about 1 month prior to current admission, in which he presented with fever, dry cough, and dyspnea. At that time, he was admitted to a general medical ward with preliminary impressions of pneumonia and diabetic ketoacidosis and received antibiotic therapy and insulin. The patient's condition improved, and he was finally discharged from the hospital after an eight‐day course. Due to prominent cough and wheezing, a short course of prednisolone 10 mg/day and inhaled steroids were prescribed at discharge. However, his cough persisted, which later aggravated and rapidly progressed to dyspnea till one night before his current hospital admission and was consequently transferred to the hospital by emergency medical services (EMS) staff. The patient suffered cardio‐respiratory arrest early in the emergency room, in which cardiopulmonary resuscitation, endotracheal intubation, and mechanical ventilation were successfully done, and was subsequently admitted to the ICU ward. Spiral chest CT scan with intravenous contrast showed a mass causing near‐total obstruction of the carina and proximal part of the right main bronchus (Figure 3) On ICU admission, peak airway pressure was persistently high, and venous blood gas revealed acute severe respiratory acidosis while Paco2 was 110 mmHg.

**FIGURE 4:**
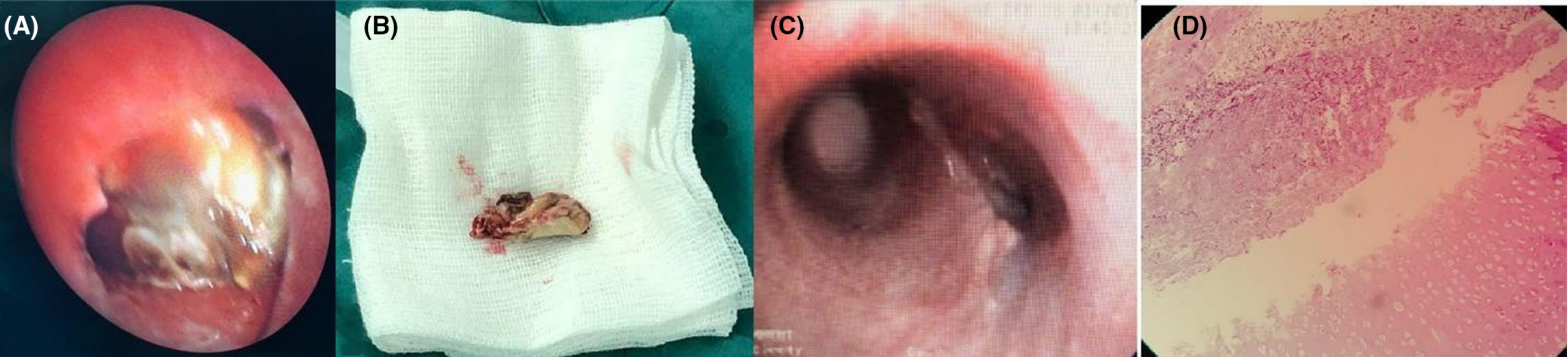
Initial bronchoscopy demonstrating (A) obstructing creamy necrotic mass in the distal part of the trachea, carina, and proximal part of the right main bronchus; (B) Gross view of the creamy necrotic mass diagnosed as mucormycosis; (C) Follow‐up bronchoscopy two months later: main carina deformity and severe right main bronchus narrowing; (D) Pathology slide demonstrating tracheal cartilage

Therefore, the following day, he underwent emergent fiberoptic and rigid bronchoscopy under general anesthesia, and the obstructing creamy necrotic mass (Figure 4A) was seen in the distal part of the trachea, carina, and proximal part of the right main bronchus. The mass was removed through rigid bronchoscopy, in which the histopathological study confirmed the mucormycosis infection involving tracheal cartilage. Liposomal amphotericin B (5 mg/kg/day) was started, and the patient was successfully extubated and transferred to a medical ward for the completion of the prolonged course of antifungal therapy.

During the next 4 months, the patient developed repeated episodes of right lung collapse due to residual right main bronchus narrowing (Figure 4C). Therefore, repeated rigid bronchoscopic dilation and electrocautery were attempted, but due to poor response to this intervention, persistent lung collapse, and post obstructive lung abscess, right lung pneumonectomy was performed. After the operation, the patient's symptoms were relieved, and he had an uneventful post‐op course in the following 6 months.

## 3 DISCUSSION

We presented two diabetic patients with non‐responding and prolonged dyspnea and cough that experienced acute respiratory failure and finally were admitted to the ICU ward because of their need for mechanical ventilation. The first patient was initially treated with broad‐spectrum antibacterial agents without a favorable clinical response. In this case, the initial improvement and weaning from mechanical ventilation in the ICU ward could be related to the improvement of concomitant bacterial infection and frequent tracheal suctioning, and possibly removal of some parts of the intra‐luminal mass. As we know, these were the first reported cases of near‐complete tracheal obstruction and respiratory failure by mucormycosis that were managed emergently with fiberoptic bronchoscopic excision of the obstructing mass. Also, mucormycosis has never been reported as a cause of difficult intubation in the ICU ward previously.

Mucormycosis is an opportunistic infection that usually involves diabetic patients (with or without ketoacidosis), cases with hematologic malignancies or organ transplantation, and patients who are on iron chelator or broad‐spectrum antibiotics. The most common form of pulmonary involvement in mucormycosis is pneumonic infiltration with variable and somewhat characteristic radiologic features.^1^ The involvement of large airways with or without parenchymal disease is less common.^1^

Tracheobronchial involvement by mucormycosis has been reported previously by others.^1^, ^2^, ^3^, ^4^ A systematic review by Ruoxi et al. reported 60 cases of mucormycosis in the tracheobronchial tree.^5^ Among them, only 10 had tracheal involvement concurrent with bronchial involvement. Furthermore, Ruoxi's review and Donahue et al.’s^6^ report confirmed that diabetic patients have a predilection for endobronchial diseases. The common feature of most reported cases is uncontrolled diabetes with or without ketoacidosis as a major risk factor.

In contrast to previous reports, our first patient's diabetes was iatrogenic and secondary to corticosteroid therapy for the treatment of primary biliary cirrhosis. It seems that hyperglycemia per se specifically pans the upper airways (including nasal and paranasal sinus) to the invasion of mucormycosis. Early diagnosis and appropriate complementary debridement or tracheostomy, as well as antifungal therapy, are critical to limit the progression of destruction of airways and improve the outcome of patients with mucormycosis.^3^

There are some clues for early diagnosis of tracheobronchial involvement. New onset of hoarseness, stridor, and significant wheezing together with fever in a diabetic patient may be helpful features. From the aspect of critical care, as in our cases, one cause of extubation failure or difficult intubation in the appropriate clinical context may be tracheal mucormycosis.

In some cases reported previously, airway obstruction was at the larynx or upper parts of the trachea, and it was finally relieved with tracheostomy or surgical debridement.^7^, ^8^ Like our described patient, Al‐Majed et al. *r*eported a 40‐year‐old diabetic man who presented with right lower lobe bronchus obstruction due to mucormycosis. They removed the fungus mass using a rigid bronchoscope.^9^ So, in an emergency situation, mainly when surgical intervention is not feasible, physical removal of the fungus mass with a flexible or rigid bronchoscope may be lifesaving. Furthermore, the value and urgency of our report should be kept in mind with the increase in the number of mucormycosis infection cases following the coronavirus disease pandemic.^10^In conclusion, mucormycosis may rarely present as near‐complete tracheal obstruction. It is possible to relieve the obstruction by fiberoptic or rigid bronchoscopy to prevent death due to asphyxia. In addition to more common causes of difficult intubation, extubation failure, or post‐extubation stridor, tracheal mucormycosis should be kept in mind in diabetic, and other susceptible patients admitted to the hospital. Also, new‐onset wheezing and stridor in uncontrolled diabetes mellitus should warrant a detailed investigation of major airways.

## AUTHOR CONTRIBUTIONS

MF designed the study and carried out the treatments. RN and BZ made a significant contribution to this study and were involved in the treatment of the patients. RS collected the data and drafted the manuscript. All authors proofread and approved the final version of the manuscript.

## FUNDING INFORMATION

No financial support was received for this case report.

## CONFLICT OF INTEREST

The authors declare that they have no competing interests.

## ETHICAL APPROVAL

Written informed consent was obtained from the patients in our study. The purpose of this research was completely explained to the patients and was assured that their information will be kept confidential by the researcher. The present study was approved by the Medical Ethics Committee of the academy.

## CONSENT

Written informed consent was obtained from the patients regarding the publication of this case report.

## Data Availability

All data regarding this study has been reported in the manuscript. Please contact the corresponding author if you are interested in any further information.
